# In vitro tooth-shaped scaffold construction by mimicking late bell stage

**DOI:** 10.3906/biy-2002-19

**Published:** 2020-10-13

**Authors:** Pakize Neslihan TAŞLI, Gül Merve YALÇIN ÜLKER, Alev CUMBUL, Ünal USLU, Şahin YILMAZ, Batuhan Turhan BOZKURT, Fikrettin ŞAHİN

**Affiliations:** 1 Department of Genetic and Bioengineering, Faculty of Engineering and Architecture, Yeditepe University, İstanbul Turkey; 2 Department of Oral and Maxillofacial Surgery, Faculty of Dentistry, İstanbul Okan University, İstanbul Turkey; 3 Department of Histology and Embryology, Faculty of Medicine, Yeditepe University, İstanbul Turkey

**Keywords:** Hydroxyapatite, stem cell, mesenchymal stem cell, differentiation, tissue engineering, hard tissue engineering, tooth scaffolding

## Abstract

Neogenesis of osseous and ligamentous interfacial structures is essential for the regeneration of large oral or craniofacial defects. However, current treatment strategies are inadequate in renewing supporting tissues of teeth after trauma, chronic infections or surgical resection. Combined use of 3D scaffolds with stem cells became a promising treatment option for these injuries. Matching different scaffolding materials with different tissues can induce the correct cytokines and the differentiation of cells corresponding to that particular tissue. In this study, a hydroxyapatite (HA) based scaffold was used together with human adipose stem cells (hASCs), human bone marrow stem cells (hBMSCs) and gingival epithelial cells to mimic human tooth dentin-pulp-enamel tissue complexes and model an immature tooth at the late bell stage in vitro. Characteristics of the scaffold were determined via SEM, FTIR, pore size and density measurements. Changes in gene expression, protein secretions and tissue histology resulting from cross-interactions of different dental tissues grown in the system were shown. Classical tooth tissues such as cementum, pulp and bone like tissues were formed within the scaffold. Our study suggests that a HA-based scaffold with different cell lineages can successfully mimic early stages of tooth development and can be a valuable tool for hard tissue engineering.

## 1. Introduction

Regenerative and multilineage differentiation (multipotency) abilities of stem cells (SCs) make them a valuable source of cells for regeneration and tissue reorganization studies. In particular, mesenchymal stem cells (MSCs) attracted researchers’ interest as a favourable source of SCs. MSCs are post-embryonic adult stem cells that can be isolated from virtually all tissue types; such as cartilage, skin, fat, dental, umbilical cord and fetal tissues (Cihova et al., 2011; Chen and Liu, 2016; Nikolova and Chavali, 2019). Upon induction, MSCs are capable of differentiating into a variety of cell lineages, such as osteocytes, odontocetes, chondrocytes, myocytes, and adipocytes under in vitro (Hauner and Löffler, 1987; Grigoriadis et al., 1988; Wakitani et al., 1995) and in vivo conditions (Benayahu et al., 1989; Bruder et al., 1998). Recent studies employed MSCs in tissue engineering, where they were used for bone, teeth, skin and cartilage repair (Djouad et al., 2009; Berardi, 2018; Han et al., 2019).

Bone marrow stroma is known to be an ideal source of MSCs, which are thought to be the main source of osteogenic stem cells, however, bone marrow derived MSCs are difficult and painful to harvest, and each harvest offers only a low number of cells. Due to these circumstances, alternative MSCs sources, such as the supportive stroma of the mesenchymal part of fat tissue, are being investigated for clinical applications. These stromal cells show characteristics of MSCs, such as plastic adherence, expression of stem cell surface markers, and the ability to differentiate into several mesodermal cell lines: bone, cartilage, muscle, epitelium, fat and neural progenitors. These adipose-derived stem cells (ADSCs) can be isolated with ease and at high quantities from patients, making them a strong candidate to be used in the field of regenerative medicine.

From its initiation of differentiation to its termination, growth of a tooth in vertebrates depends on cell-to-cell interactions between the dental epithelium and mesenchyme (Puthiyaveetil et al., 2016). These interactions include a variety of signaling networks that are composed of different signaling molecules, their receptors and expressional control systems. Most of these signaling molecules are growth factors: such as bone morphogenetic proteins (BMPs), fibroblast growth factors (FGFs), and factors from Wnt, and Hedgehog (Hh) families. Tooth allo-auto transplantations, and dental implants are in widespread use in dentistry as a tooth replacement option for many years, and MSCs mediated tissue engineering for replacement organs and tissues for these clinical applications has become very promising (Aberg et al., 2004).

HA makes up around 60% bone and enamel of teeth are by weight. As a result, HA has been investigated intensively as a component of hard-tissue scaffolds (Oonishi et al., 2000). In vitro and in vivo studies suggest that HA induces the attachment, differentiation and proliferation abilities of stem cells. Mechanical properties of tooth, such as its crystallinity, porosity, and composition change with the development of the body. HA based scaffolds mimic such changes as time passes, making it an ideal scaffold.

**Figure 1:**
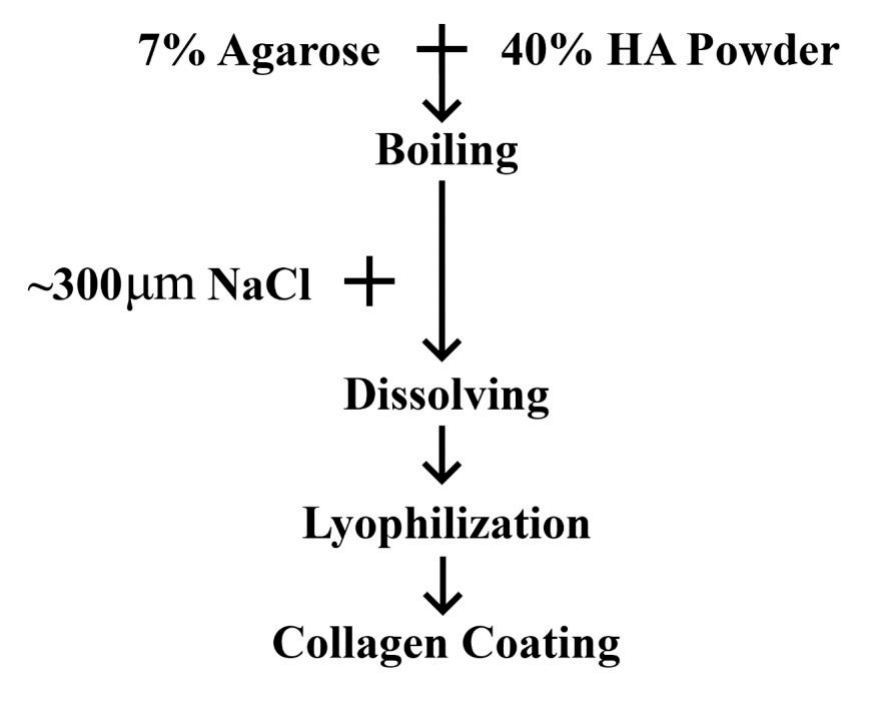
A flow chart of process steps for scaffold fabrication using a combination of agarose and NaCl.

Mimicking teeth at the late bell stage for hard tissue scaffolds can be difficult, as it requires a combination of structures such as dentin, ligament, enamel, pulp and cementum. While the mechanical and biological effects of HA in hard tissue scaffolds are known (Chu et al., 2004), a combination of HA with bone marrow MSCs, neural crest derivative adipose MSCs and ectoderm derivative epithelium have not been studied. Previous studies have shown that all components of teeth, such as enamel, dentin and pulp, must be fully formed to form functional teeth. This study attempts to create a functional tooth with a combination of the physical, tooth-shaped porous HA scaffold with cellular components of epithelial cells, hADSCs and hBMSCs (Figure 1).

## 2. Method

### 2.1. Preparation of tooth-shaped scaffolds and cell seeding

The tooth-shaped scaffolds were prepared using agarose and HA, with a novel method that integrates the gel-casting and salt dissolving methods. 7% (w/v) agarose (Sigma-Aldrich Chemie GmbH, Taufkirchen, Germany) and 40% (w/v) HA (Sigma-Aldrich Chemie GmbH) was dissolved in dH2O via boiling. Before the solution completely cooled off, 300 µM of NaCl (Sigma-Aldrich Chemie GmbH) was added to a concentration of 50% (w/v) to the HA slurry. After polymerization, NaCl was removed from the HA scaffold by immersing it in dH2O for 24 h. Afterwards, the scaffolds were lyophilized. Constructs were shaped into human molars at the late bell stage. Tooth-shaped scaffolds were then coated with collagen at 4 °C overnight, washed with DMEM (Gibco, Thermo Fisher Scientific Inc., Waltham, MA, USA), and seeded with 2 × 106 cells suspended in DMEM for 1h at 4 °C (Figure 1).

### 2.2. Scanning electron microscopy

Scanning electron microscopy (SEM) was used to characterize the structural nature of the HA scaffolds. HA scaffolds with or without human ADSCs were subjected to 3% glutaraldehyde, 0.1 M sodium cacodylate and 0.1 M sucrose for 1 h. Fixed samples were dehydrated using sequential concentrations of alcohol, and coated with gold using a sputter coated (BAL-TEC SCD 005, BalTec Maschinenbau AG, Pfäffikon, Switzerland). SEM images of the samples were taken using SEM Zeiss EVO 40 (Carl Zeiss Microscopy GmbH, Oberkochen, Germany).

### 2.3. Porosity and density measurements

Porosity and density of the HA scaffolds were determined via their liquid replacement. Structural properties, such as porosity, pore sizes, their distribution, scaffold permeability and presence of physical deformities were determined through physical measurements. Briefly, an HA scaffold of known weight (W) was immersed in a graduated cylinder containing a known volume (V1) of ethanol (EtOH). The cylinder was then placed under vacuum to force EtOH into the pores completely, indicated by the end of any bubbles escaping the scaffold. Volume of the HA scaffold was measured by subtracting the initial volume V1 from the final volume after immersion, V2. Next, the scaffold was removed without spilling any HA within the scaffold. Inner volume of the scaffold V was then calculated as:

V = V2 - V3

The measured density of the scaffold, p was calculated as

P = W / (V2 - V3)

The porosity of the scaffold, m was calculated as

m = (V1 - V3) / (V2 - V3)

### 2.4. Infrared spectroscopy

Characterisation of powdered HA was done using infrared (IR) spectroscopy. Polarized Fourier transformation infrared (FTIR) of HA, with a depth of penetration of 2 µm at 1000 cm–1 was obtained using Thermo Scientific Nicolet™ iS™ 10 FTIR spectrometer coupled with attenuated total reflectance (ATR) sampling accessory and solid transmission sample compartment. Resulting spectra were analyzed using standard Nicolet iZ10 and OMNIC software. Both HA nano powder and HA scaffold were analyzed using FTIR.

### 2.5. Cell culture

Immortalized human gingival keratinocytes (Gie-No3B11) cells were purchased from ABM Industries Inc. (New York, NY, USA) and adipose-derived mesenchymal stem cells; normal, human (PCS-500-01), bone marrow-derived mesenchymal stem cells; normal, human (PCS-500-012) cells were purchased from American Type Culture Collection (ATCC, Manassas, VA, USA).

### 2.6. In vitro odontogenic induction

Odontogenic differentiation medium was prepared media containing 50 μg/mL ascorbic acid (Sigma-Aldrich Chemie GmbH; Merck KGaA, Darmstadt, Germany), 50 nM β-glycerol phosphate (Sigma-Aldrich Chemie GmbH, Merck KGaA), and 10−8 M dexamethasone (DXM) (Sigma-Aldrich Chemie GmbH, Merck KGaA). Cells were treated with the induction medium for 14 days, changing the media every other day. Positive control groups received differentiation medium, while the negative control cells received just DMEM.

### 2.7. Alkaline phosphatase (ALP) activity

ALP activity of cells was measured after odontogenic induction. Briefly, media was removed, and 0.2% (w/v) Triton-X-100 (Sigma-Aldrich Chemie GmbH; Merck KGaA) were added for cell lysis. Cells were harvested from 6-well plates, and vortexed for 20 min at room temperature for mixing. 10 μL of lysate was then added to wells of a 96-well plate, and then 90 μL of ALP solution (Abcam PLC, Cambridge, UK) was added to lysates. Absorbance was measured by ELISA plate reader to determine ALP activity.

### 2.8. Alizarin red staining

Alizarin red assay measures free calcium or calcium deposits of cells. Cells were seeded in 6-well plates at a concentration of 50,000 cells/well. After the differentiation process, cells were fixed with 500 μL of 2% paraformaldehyde (Sigma-Aldrich Chemie GmbH; Merck KGaA) for 20 min at room temperature, rinsing thrice with PBS (Gibco, Thermo Fisher Scientific Inc., Waltham, MA, USA) at the end. Afterwards, 3% (w/v) silver nitrate (ScienCell Research Laboratories, Inc., Carlsbad, CA, USA) was added, and the cells were incubated under UV light for 1 hat room temperature. Cells were rinsed with PBS thrice, and 5% (w/v) sodium thiosulfate (ScienCell Research Laboratories, Inc.) was added into each well, followed by a 2 min incubation at room temperature. Colour formation was observed under a light microscope.

### 2.9. Quantitative real-time polymerase chain reaction

Total RNA isolation was completed using the High Pure RNA isolation kit (Roche Diagnostics International AG, Rotkreuz, Switzerland) according to the manufacturer’s instructions. RNA samples were translated into cDNAs with High Fidelity cDNA synthesis kit (Sigma-Aldrich Chemie GmbH; Merck KGaA). Gene levels were evaluated by real-time PCR using Maxima SYBR Green kit (Fermentas, Thermo Fisher Scientific Inc.). Synthesized cDNAs were used as a template for gene level analysis. Odontogenesis related genes: BMP4, COL1A, DSPP and as a housekeeping gene GAPDH was used.

### 2.10. ELISA

Levels of FGF 3, FGF10and BMP4 proteins in cell supernatants were determined with sandwich ELISA (RayBiotech, Inc., Peachtree Corners, GA, USA) according to the manufacturer’s instructions. Briefly, for each protein; standard proteins and samples collected from media culture were added to antibody coated plates and incubated overnight at 4 °C. Biotinylated detection antibodies were added to the plates, and incubated at room temperature for 2 h, followed by incubation with peroxidase-labelled streptavidin. Absorbance was measured at 490 nm by ELISA plate reader (BioTek Instruments, Inc., Winooski, VT, USA) to determine protein expression levels.

### 2.11. Histopathology

After 8 weeks, developing tooth-shaped scaffolds were excised, fixated with 4% formalin, embedded in paraffin and sectioned into thin sections. Samples were collected at weeks 0th, 4th, and 8th and stained with hematoxylin and eosin Y (H&E) staining, and Masson’s trichrome staining.

For Masson’s trichrome staining, sections were deparaffinized by incubating them at 65 °C, and then treated with xylene and EtOH dilution for dehydration. Next, the sections were rinsed with dH2O and placed into solution A, consisting of acid fuchsin, dH2O and glacial acetic acid for 10 min followed by a rinse of tap water. Then, the sections were placed into solution B, consisting of phosphomolybdic Acid and dH2O, for 5 min followed by a rinse of tap water. Finally, the sections were placed into solution C, consisting of methyl blue, glacial acetic acid and dH2O for 10 min and rinsed well with dH2O. Water was then removed with EtOH and xylene.

For H&E staining sections were deparaffinized by incubating them at 65 °C. Sections were then treated with xylene and EtOH dilution for dehydration. Next, the sections were rinsed with dH2O and placed into hematoxylin solution for 3.5 min, rinsing twice with water at the end. Next, sections were placed into a solution 1% of acid alcohol for 1 min followed by a rinse with water. Sections were quickly dipped into and then removed from an ammoniac solution, and rinsed with water. Sections were placed into 96% EtOH solution for 30 s, and then placed into Eosin Y solution for 6 min. Samples were dehydrated with serial EtOH dilutions, and cleaned with xylene.

### 2.12. Immunohistochemical analysis

Immunohistochemical analysis were performed to characterize cellular morphology of immature tooth at the late bell stage with bone morphogenetic proteins 2, 4, 7 (BMP 2, 4, 7), amelogenin, collagen type 1 (COL1A), dentin matrix protein 1 (DMP 1), dentin sialophosphoprotein (DSPP), vascular endothelial growth factor (VEGF), CD 31 and Msx 1. Immunohistochemical staining to tooth-shaped scaffold at the 0th, 4th, and 8th weeks were performed on paraffin sections.

### 2.13. Statistical analysis

Statistical analysis of the results was done using GraphPad Prism5 software. For statistical analysis, Student’s t-test was. P values less than 0.05 were considered statistically significant. All figures were prepared using the Microsoft Office Excel and GraphPad Prism5 softwares.

## 3. Results

### 3.1. Porosity and density measurements

The density of a porous scaffold can influence its physical strength, permeability, and presence of structural defects. Table shows the density and porosity of HA scaffolds prepared with different concentrations of HA. Characterisation of the scaffold porosity was measured through scaffold permeability and surface area of its porous structure. More porous structures have a higher surface area to volume ratio, which is favourable for attachment of cells and for altered hard tissue regeneration. Table shows scaffolds that with approximately the same porosity but higher HA content had higher densities. Increasing the HA concentration led to scaffold with pores that were interconnected with denser and thicker pore walls – factors that improve the mechanical properties of the scaffold.

**Table:** Table. Density and porosity values at changing HA concentrations.

HA concentration	Pore size	Density g/cm3	Porosity %
35 % (wt/vol)	150–300 µm	0.043 ± 0.002	84.2 ± 0.3
40 % (wt/vol)	150–300 µm	0.048 ± 0.002	91.5 ± 0.3
45 % (wt/vol)	150–300 µm	0.051 ± 0.002	90.0 ± 0.3
50 % (wt/vol)	150–300 µm	0.055 ± 0.002	87.1 ± 0.3

### 3.2. Odontogenic differentiation of hADSCs

ADSCs were seeded for a 2 weeks odontogenic induction. Odontogenic differentiation of ADSCs were evaluated by measuring calcium deposition with alizarin red staining. Supplementary data shows Ca deposits on differentiated cells (left), negative control shows Ca deposition and odontogenic induction (right). Alkaline phosphatase (ALP) activity measurements were performed to calculate differentiation levels (Supp. data 1). COL1A and DSPP gene levels were measured by RT-PCR. These genes play an important role of hard tissue formation and dentinogenesis (Supp. data 1). DSPP and COL1A responsible for dentinogenesis and extracellular matrix formation during maturation states of dentinogenesis (Wahl and Czernuszka, 2006; Zhu et al. 2012).

### 3.3. SEM images of scaffold

**Figure 2:**
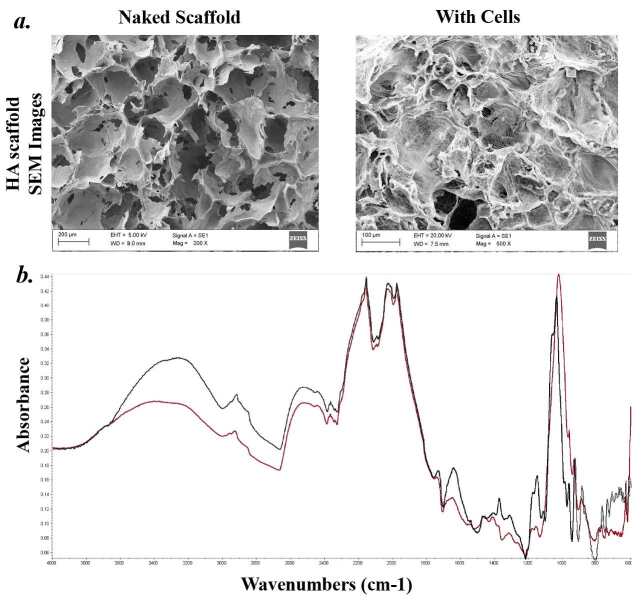
SEM images show the porous structure of HA hybrid scaffold with open and connective form. SEM images of interconnected porous HA scaffold prepared with mixture containing 40% (wt/wt) HA with and without cells. FTIR spectra of HA scaffolds. Figure shows the difference between (black line) HA powder and (red line) scaffolded HA.

Tissue scaffolds should mimic their target tissue in both composition and physical characteristics. Porous scaffolds aid in the development of tooth and its soft tissues within the large pores, and allow for proper blood supply for further hard tissue mineralization. SEM images of the scaffolds in Figure 2a show the macroporous interconnected nature of HA scaffolds with and without cells. Pore sizes of scaffolds were measured between 100 μm and 300 μm. All samples showed the basic structural characteristics of a continuous open structure, with a 3D network of interpenetrating struts and pores. Hubert et al. suggests that pore sizes between 150–200 µm are optimal for osteoconduction. In the IR spectra, peaks of 630 and 3570 cm–1 correspond to the vibration of OH ions, 1030 and 570 cm–1 are the typical peaks of phosphate bending vibration, while the peak at 985 cm–1 is known to be phosphate stretching vibration. The peaks at 881, 1420 and 1460 cm–1 are indicative of the carbonate ion substitution. The peaks at 1650 and 3470 cm–1 correspond to H2O absorption (Figure 2b). Spectra of HA powder and scaffold had no observable differences, confirming that the scaffolding processes do not alter the chemical composition of HA.

### 3.4. Cross talk between cells on the scaffold

**Figure 3:**
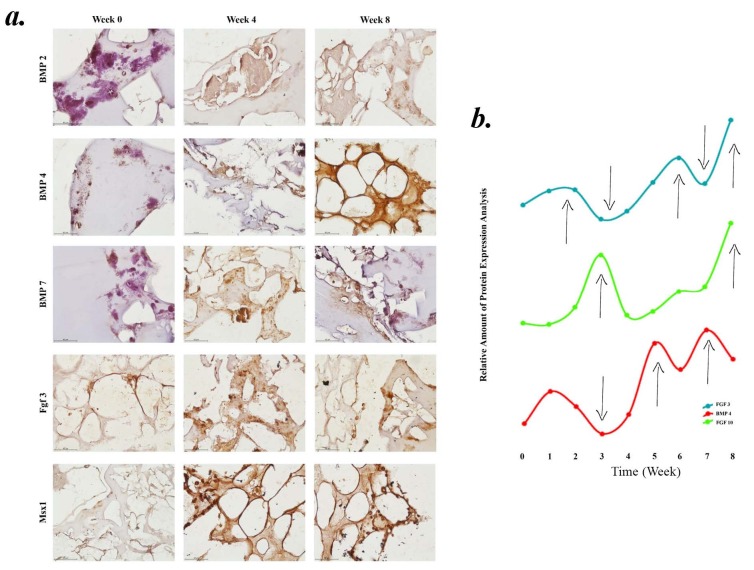
(a) Time dependent immunohistochemical staining of tooth forming cells. Used antibodies are BMP 2, 4, 7, FGF 3 and Msx 1, (b) relative FGF 3, FGF 10 and BMP 4 proteins released from cells seeded on tooth-shaped scaffolds for 8 weeks.

BMP 2, BMP 4 and BMP 7 are important players of the signaling network that regulates the initiation of teeth growth and development. Cells seeded to the HA scaffold initially (Week 0) expressed BMP2 from epithelial tissues, transforming the tooth into a bell shape. At the early bell stage (Weeks 4 and 8), BMP2 expression disappeared and shifted from epithelial tissues to the central cells of the dental papilla mesenchyme (Week 8). Subsequently, expression extended coronally in the dental papilla and differentiates the cells to odontoblasts (Figure 3a). A similar expression pattern was observed with BMP7, where the transcripts were localized transiently in both ameloblast and odontoblast portions, and was then down regulated as development advanced. The expression of BMP4 disappeared from the dental epithelium after the removal of the enamel knot (Weeks 0–4), and expressed in the area between epithelium and differentiated odontoblastic cells (Week 4). At the 8th week BMP4 expression shifted to the dental mesenchyme part. The epithelial cells showed very weak or no expression (Week 8). Msx 1 expression was controlled by BMP 4. Msx 1 expression was triggered at the dental mesenchyme by increment of BMP4 expression in the dental mesenchyme cells. At the late stage of cap and early stage of tooth development enamel knot, formed by epithelial cells, expresses the FGF 3 (Week 0). At the middle of the bell stage enamel knot started to disappear and FGF 3 expression was observed at dental mesenchymal cells, mainly odontoblasts.

The Cells were seeded on porous HA scaffold and treated with odontogenic media for 8 weeks. Media was collected every week to measure secreted cytokines and growth hormones. Figure 3b shows FGF 3, FGF 10 and BMP 4 released from differentiated cells. FGF3 and FGF10 expressed from dental mesenchyme at the bud stage. While FGF10 can stimulate cell proliferation, it can only do so in the epithelium but not in the mesenchyme; FGF3 can stimulate cell proliferation in isolated mesenchyme (20). BMP 4 is expressed from enamel knot, and may be cause for apoptosis in the knot cells at the end of the late bell stage and start to express in the dental mesenchyme.

Secretion profile of FGF3 and FGF10 in the 1st weeks were not parallel, which shows the cross-talk between epithelial and mesenchyme cells. Initially, at the 3rd week, FGF10 expression increased and proliferated epithelial cells, forming the enamel knot. Meanwhile FGF 3 and BMP 4 release decreased. At the 4th week, the enamel knot started secreting BMP 4, which triggers proliferation of the dental mesenchyme and their differentiation. At the 7th week, enamel knot starts to release FGF 3 to alter dental mesenchyme and start dentinogenesis. During the last week, cells differentiated and started forming dentin and enamel.

### 3.5. Histological examination of tooth-shaped scaffold

**Figure 4:**
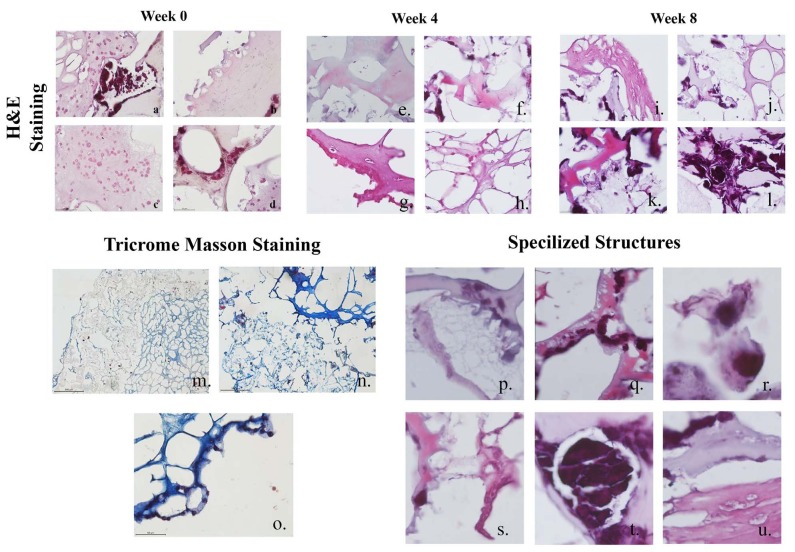
H&E staining of 0th, 4th, and 8th week tooth-shaped grafts, Masson’s trichrome staining of 8th week tooth-shaped grafts and specialized structures of cells seeded on HA hybrid scaffolds for 8 week.

At the 0th, 4th, and 8th weeks, scaffolds were stained with H&E (Figure 4). Disoriented cells (Figures 4a–4d), and mesenchyme derived odontoblastic cells (Figures 4a and 4c–4d) were observed on the scaffolds. The scaffold had a non-degraded porous structure (Figure 4d). At the 4th week, cells on the scaffold started to localize and differentiate, and cells started the degradation of the scaffold. Formation of predentin (Figure 4e), low level of dentin (Figure 4f) differentiated odontoblasts (Figure 4g) and pulp matrix formation (Figure 4h) were observed at this point. At the 8th week, mature cells and structures such as cementoblast cells were observed forming into an outer mesenchyme (Figure 4i), leading to cellular communications between differentiated epithelial and odontoblasts, expression of dentin and enamel precursors (Figure 4j), formation of epithelial cells into the mature dentin (Figure 4k) and differentiated, specialized epithelial Malassez (Figure 4l).

Collagen containing sections of the scaffold are stained by Masson’s trichrome. Collagen secretion mainly occured at the pulp section (Figures 4m and 4n) and odontoblast cells (Figures 4n and 4o). Figure 4 shows specialized structures for tooth development.

First, cell morphology shifted to be cementocyte like (Figure 4p) and cementoblast started to form in the outer mesenchyme (bone marrow stem cells) (Figure 4q). Dentinogenesis started at this stage (Figure 4q). Odontoblasts started to differentiate and secrete dentin (Figure 4r). Purple regions indicate epithelial cells, their opposite region is surrounded by odontoblasts, and pink regions indicate the dentin (Figure 4s). After the 4th week enamel knot started to appear (Figure 4t). Enamel knots are created by specialized epithelial cells, which play important roles during the period of teeth growth before mineralization. Figure 4u shows differentiation of epithelial cells, which start forming into an elongated morphology, and start communicating with the dentin, forming odontoblasts that form into the enamel surface. 

### 3.6. Late odontogenesis

**Figure 5:**
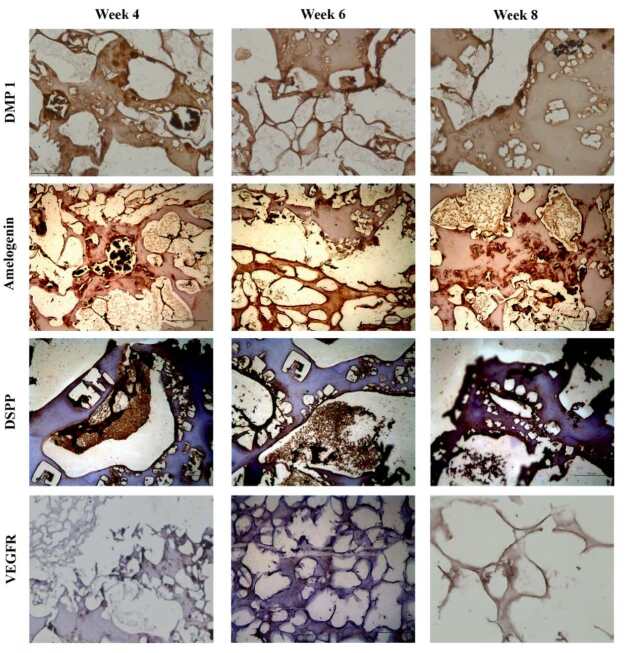
Time dependent immunohistochemical staining of tooth forming cells. Used antibodies are DMP 1, DSPP, amelogenin and VEGFR.

Expression of DMP 1, amelogenin and DSPP only occurs in mineralized tissues by differentiated cells after they are induced by the cellular communications between epithelial and mesenchymal cells. Even though DMP 1 is not necessary to early tooth development (Week 0), its expression is necessary for mineralization and formation of the proper teeth structure at later stages (Weeks 4–8) (Figure 5). VEGF is the key regulator in endothelial cells for the development of blood vessels. VEGF expression was detected at both the dental mesenchyme and the epithelial sections, which were formed into blood vessels like morphology (Weeks 4–8).

## 4. Discussion

Interest in the use of interconnected microporous 3D scaffolds to mimic the growth of various organs, surrounding tissues, vascularization and tissue remodelling has increased over the last decade (Chen and Liu et al., 2016; Fu and Wang, 2018; Turnbull et al., 2018; Nikolova and Chavali, 2019; Sarker et al., 2019). Especially, the use of homogenous interconnected porous scaffolds for hard tissue engineering promotes vascularization and intercellular communications between different cells, which leads to the formation of complex, 3D tissues with correct cells and extracellular matrix components. Creation of these porous scaffolds can be done rapidly with the gel casting method, converting ceramic particles into scaffolds with desired form and porosity through in situ polymerization, which creates a macromolecular network that fuses the ceramic particles together (Sepulveda et al., 2000; Netz et al., 2001). Neither the gel component nor ceramic particles of the scaffold are toxic to the cells, and the cells are capable of removing the biochemical gel component via biodegradation, allowing these materials to be used in hard tissue engineering studies (Sepulveda et al., 2000). Advantages of gel casting are the rapid gelation of the casting material, which prevents accumulation of the blend and results in a homogeneous polymer matrix with equal particle dispersion, and a desirable size of macroporous HA structure. Modifications to the size and geometry of the pores can be achieved by modifying the size of NaCl particles. Figure 2a show the porous, interconnected and uniform microstructure of HA scaffolds. Scaffolds prepared with four different HA concentrations, 35%, 40%, 45% and 50% (wt/wt), were used to observe the effects of HA concentration on physical features of scaffolds. Even though a higher HA concentration leads to a harder, denser HA scaffold, solutions with high concentrations of HA have a high viscosity, making the formation of a more homogeneous. Hence, a concentration of 50% (wt/wt) HA was selected as the highest concentration to be used in this study. Biocompatibility of hard-tissue scaffolds are affected by the scaffolds composition (Eggli et al., 1988; Best et al., 1997). Cellular response to the scaffolding material changes with different ratios of calcium and phosphorus, which change the solubility and mechanical strength of the final scaffold (Best et al., 1997). Figure 2a shows SEM images of the macroporous HA scaffolds prepared with 40% (wt/wt) HA nanoparticles in the blend. Spectra of the materials were measured with IR spectroscopy before and after the formation of the HA scaffolds. FTIR spectra results, as shown in Figure 2b, show two peaks at 630 and 3570 cm–1 which correspond to the vibrations of OH ions, 1030 and 570 cm-1 are the typical peaks of phosphate bending vibrations, while the peak at 985 cm-1 is known as phosphate stretching vibrations. The peaks at 881, 1420 and 1460 cm-1 are indicative of the carbonate ion substitution. The peaks at 1650 and 3470 cm-1 correspond to H2O absorption. The results show that there were no discernible spectrum differences between HA nanoparticles and constructed HA scaffold, as seen in Figure 2c. Shaped scaffolds must mimic the properties of natural teeth, both with chemical composition, and with physical morphology and characteristics. The scaffolds porous structure promotes development of soft and hard tissues with proper circulation within the pores, which leads to further vascularization and mineralization of the related tissues. SEM images of the interconnected, macroporous architecture of the HA scaffolds with pore sizes ranging between 150–300 µm can be seen in Figure 2a. Prepared HA scaffolds display an uninterrupted open porous structure, with a 3D interpenetrating network of struts and pores. While the pore sizes of the HA scaffolds are over the suggested ~150 µm by Hulbert et al. (1972), Flatley et al. (1983) has shown that pore sizes of ~500 µm are suitable for osteoconduction. Pore sizes between 150–300 µm, and an interconnecting pore structure like the ones shown in Figure 2 are ideal for odontoblasts, epithelial cells and osteoblasts to expand into the scaffold pores and cavities (Joschek et al., 2000), for fast vascularization, teeth generation, cellular interactions and remodeling (Holmes, 1979; Schliephake et al., 1991). Rapid and organized vascularization of the tissues and the interconnected pore structure are also necessary for tissue development. The porous scaffold serves as a supporting material for the forming hard tissue, but also allows secreted molecules and degraded monomers to freely move through the scaffold. HA density of a porous scaffold affects its mechanical strength, permeability, but may also lead to structural defects (Flatley et al., 1983). Density and porosity of HA scaffolds prepared with blends of different HA concentrations were shown in Table. One challenging factor of the hard tissue engineering is identifying the proper pore size and pore amount for the application. Porosity of the scaffold affects its permeability and surface area. Highly porous scaffolding with low HA concentrations resulted in higher surface area/volume ratio and a higher cell attachment rate, but the scaffolds had a lower mechanical strength. On the other hand, highly porous scaffolds with high HA concentrations had thicker and denser pore walls that originated from a stable, nondisrupted coating and contained fewer defects, which resulted in a structure strong in mechanical strength. Although some progress has been reported on scaffolding built to support dental tissue, replicating certain stages of teeth development ex vivo remains a challenge, let alone the regeneration of the three phase enamel-dentin-pulpa (De Groot, 1980). For the functional regeneration of the developed teeth constructs, cells of different origins, proper extracellular matrix components, fibrous connective tissues require orientation towards bone surfaces to allow optimal biomechanical integration. In this in vitro study, a new methodology consisting of cell cultures, 3D scaffolding and HA nanopowder were engineered together to form a scaffold capable of growing a complex tooth. The controlled 3D porous structure of the scaffold mimicked the nature and anatomic structure of immature teeth at the late bell stage. Different cell types present in the formation of teeth in vivo were represented by seeding cells of those origins separately to their corresponding positions within the scaffold. The combination of the HA scaffold and complex cellular interactions allows increased generation capacity of functional early stage of tooth within in vitro conditions.

Main difficulty of using 3D scaffolding architectures is allowing cellular interactions between different cell types, and allowing the movement of scaffolding monomers and extracellular matrix components responsible for cell migration, differentiation, reorganization, vascularization and structuralization in tissue formation or wound healing (Ramay and Zhang, 2003). Cell and tissue compartmentalization is necessary to coordinate the formations of multiple tissue complexes of teeth into a single system. Therefore, the main benefits of 3D hybrid scaffolds are the increases in morphogenesis, organization, and functionalization of multiple tissue interfaces (Ramay and Zhang, 2003). The biological contraction of dental tissues against each other was histomorphometrically observed with calcified tissue layer deposition (Figures 4a–4l) at 0, 4 and 8 weeks. In the microporous structures of the HA construct, the cells had the ability to form teeth like structures by with cell-to-cell and cell-to-microenvironment interactions, which leads to sustained enamel-dentin-pulpa formation. The 3D and multicellular scaffold system allows multitissue evaluation of two distinct mineralized structures, connected via an intervening structure that allows biomechanical simulations. This model system allows various biomechanical stimuli to be transmitted directly via mineralization or pulp induction alone. During the early stages of teeth growth, the expression a series of specific molecular markers, BMP 2, BMP 4, and BMP 7 started from the enamel knot, which acts as the signaling center of teeth development. Recent studies show the role of BMP 4, where it was given to dental mesenchyme, and lead to morphological changes and induction expression of transcription factors, including Msx 1, Msx 2, Lef 1, and Egr1 in the dental mesenchyme. The expression of BMP 2, BMP 4, and BMP 7 from enamel knot may cause apoptosis in the knot cells at the end of the late bell stage. FGF 3 is expressed from dental mesenchyme at the bud stage and stimulates cell proliferation in isolated mesenchyme (Kettunen et al., 2000). Studies showed that FGF 3 deficient mice did not exhibit important tooth defects (Mansour et al., 1993; Min et al., 1998). Msx1 regulates the expression of Cbfa1/Runx2 (Zhang et al., 2003; Åberg et al., 2004), while Msx1 also regulates FGF 3 expression from the dental mesenchyme (Bei and Maas, 1998). Msx 1 regulates Cbfa1/Runx2 expression controlling FGF 3 expression. All these transcription factors regulate the expression of growth factors that are responsible for the activation and regulation of other signaling molecules of developing teeth. During the mineralization stage, formation of the crown happens through dentinogenesis and amelogenesis, via the deposition of DMP 1, DSP and COL1A (dentin proteins) and amelogenin (enamel protein) and calcification starts at the preamelobasts, from the inner enamel epithelium, induce adjacent cells of the dental papilla to become odontoblast that then produce the dentin matrix which in turn induces the preameloblasts to become ameloblasts that produce enamel matrix. HA represents a great inductive material for hard tissue engineering. HA has a high level of biocompatibility and osteoconductivity which are shown through the expression levels of OCN and SPARC proteins, which indicates the degree of tissue maturation and stability of the teeth structures formed within the scaffold. Therefore, through these markers, the maturation and refunctionalization of generated dentin related organizations could be tracked and quantified through the marker expression levels at the generated regions (Figures 4i–4o). The biomimetic architecture achieved here provides a promising platform for dental tissue engineering. The microporous scaffold with certain cells can induce the functional integrity of generated dental complexes. While the main attraction of this study was generation of dental complexes, internal geometries and the biomimetic design can be applied to a diverse range of clinical applications. The current engineered hybrid scaffold systems still have limitations compared to the highly complex native multitissue formation (Sackstein, 2011), especially for the generation of mature teeth (Koch, 1967; Alhadlaq and Mao, 2003; Wang and Feng, 2017). In hard tissue engineering, the main challenge is the lack of a physically constructed environment mimicking the in vivo environment, and functional design that does not completely mimic the irregular and complex morphology of developed teeth (Yamaza et al., 2010). Biological advantages demonstrated in this present study using a novel, hybrid scaffold show that using a tooth-relevant design that mimics the late bell stage of teeth and respecting the anatomical specifications of teeth can improve in vitro hard tissue engineering (Figures 4p–4u).

The scaffold was designed in a modular fashion – with two separate components for the pulp and the dentinoenamel, which allows the scaffold to be easily placed into an existing tooth socket for future applications. The adaptability of the constructed scaffold was evaluated by IHC analysis to establish the tooth formation. The designed dental engineered scaffold was initially designed to increase angiogenic development and enhance biological molecule diffusivity at the 4th week (Figure 3). This concept was supported with the in vitro findings, which reflect an increased number of vascular structures, enamel, dentin and mature odontoblasts along the mineralization scaffold architecture (Figures 4p–4u). Many differentiated and interconnected groups of cells were observed at the 8th week (Figures 4i–4o.). However, by the 8th week, further mineralization caused further tissue orientation, as observed indifferent proportions for 4th week and 0th week (Figures 4a–4l). These groups of cells within the scaffold were collectively oriented along the tooth-shaped scaffold and at the center of more mature odontoblastic structures. These results also support that the hybrid scaffold design with biomimetic architecture influences cell behavior and tissue orientation towards teeth development. The cells that differentiate after the epithelial-mesenchymal transition process begin to form minerals. In this process, the expression of DMP 1, amelogenin and DSPP starts to increase with the mineral formation (Figure 5). DMP1, which is not synthesized in high amounts at the beginning of organogenesis, is an essential protein for maturation and mineralization in late stages.

Therefore, a combinational system of tooth (mesenchymal stem cells-epithelial cells) and odontogenic stimulation can enhance the generation of the multilayered tooth complex. According to our results, cementum, dentin, pulp and enamel like structures were produced, but at a micro scale within each pore, instead of a singular large tooth. Therefore, enamel, dentin or pulp forming structures should be nonporous for tooth engineering. These findings are important as they emphasize the potential biological effect of the hybrid scaffold system.

## 5. Conclusion

Combination of a gel-casted, HA-based scaffold with multiple cellular lineages in a hybrid system forms an accurate in vitro model of early tooth development. Scaffolds consist of pores ranging between 150–300 µm in an interconnected macroporous structure suitable for tissue development. Overall results showed that the scaffolding process and its product as a tooth-shaped HA scaffold are suitable for cell attachment and growth which is required for effective hard tissue engineering approaches. Problems arising from a porous scaffold should be eliminated by using a system that integrates dentin and enamel uniformly instead of a porous and harsh scaffold. In conclusion, this study demonstrates that the formation of enamel-dentin-pulp interfaces in vitro requires 3D scaffolds structured as an immature tooth at the late bell stage of development. As a future prospect, in order to eliminate these problems, HA may be mixed with highly collagenese gel or other dentin related extracellular proteins. Methodology provided in this study can be a valuable stepping stone for future in vivo studies and clinical applications.
